# Presence of late gadolinium enhancement in Duchenne muscular dystrophy patients is associated with age and global ventricular function

**DOI:** 10.1186/1532-429X-14-S1-P193

**Published:** 2012-02-01

**Authors:** Kan N Hor, Linda Cripe, Peace Madueme, Thomas D Ryan, Wojciech Mazur, Hussein Al-Khalidi, Subha V Raman, Joshua Sticka, John L Jefferies, D Woodrow Benson, Michael Taylor

**Affiliations:** 1 Pediatric Cardiology, Cincinnati Children's Hospital Medical Center, Cincinnati, OH, USA; 2 The Heart and Vascular Center at The Christ Hospitals, Cincinnati, OH, USA; 3 Duke University School of Medicine, Durham, NC, USA; 4 Ohio State University, Columbus, OH, USA

## Background

The Duchenne muscular dystrophy (DMD) associated cardiac disease results in significant morbidity and mortality usually resulting in death by the second to third decade of life. End-stage cardiac pathology consists of alternating areas of myocyte hypertrophy, atrophy and fibrosis manifesting as late gadolinium enhancement (LGE) on cardiac magnetic resonance imaging (CMR) evaluation. There has been considerable interest in detecting the presence of LGE, and previous studies have associated LGE with global ventricular dysfunction as assessed by ejection fraction (EF). However, given the genetic origins and underlying pathogenesis of DMD-associated cardiac disease, we hypothesized that LGE is prevalent in patients without overt disease.

## Methods

CMR studies performed between June 2006 and June 2011 were analyzed. Standard imaging sequences included cine images and LGE sequence of the left ventricle. Global ventricular function was assessed using QMASS® and LGE was assess qualitatively and reported as negative or positive. The presence of LGE was compared to age and EF. Statistical analysis was performed using SAS (version 9.2; SAS Institute Inc, Cary, NC) and reported as percentages and odds ratios (OR) with confidence interval (CI).

## Results

**Figure 1:**
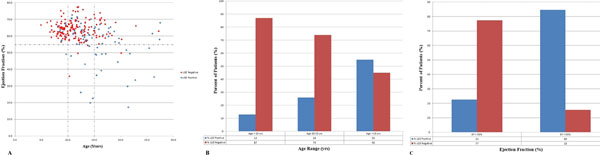
Age and EF Predicts LGE. (A) Scatter graph showing that LGE correlates highly with Age and abnormal EF (Red Square) LGE negative and (Blue Diamond) LGE positive. (B) Bar graph showing increasing LGE with age. (C) Bar graph showing higher percent of LGE positive patients when EF is abnormal.

We analyzed 247 CMR studies in individual DMD patients and compared the presence of LGE to age and EF (Figure 1A-C). The presence of LGE was strongly associated with EF; LGE was present in 85% of patients with EF < 55% whereas only 15% of those with normal EF were LGE positive (OR = 18.8, CI 6.2-57.1). LGE presence was also strongly associated with age; LGE was present in only 13% of those < 10 years (OR = 1), in 26% of those 10-15 years (OR = 3.4, CI 1.8 - 6.7) and in 55% >15 years of age (OR = 8.0, CI 3.4 - 18.6) (Table 1).

**Table 1:** LGE Compared to Age and Ejection Fraction

Parameter	Number of Pts	LGE Negative	LGE Positive	Odds Ratio	P value
Age < 10 yrs	75	65 (87%)	10 (13%)	1	
Age 10-15 yrs	114	84 (74%)	30 (26%)	3.4 (CI 1.8-6.7)	0.0003
Age > 15 yrs	58	26 (45%)	32 (55%)	7.7 (CI 3.4-18.6)	0.0001
EF > 55%	221	171 (77%)	50 (23%)	1	
EF < 55%	26	4 (15%)	22 (85%)	18.8 (CI 6.2-57.1	0.0001

EF = Ejection Fracion,LGE = Late Gadolinium Enhancement, Pts = Patients, Yrs = Years

## Conclusions

This large series shows a high prevalence of LGE in DMD patients. LGE is strongly associated with age and global dysfunction. Thus it is most common in older patients with reduced EF. However, it is also detected in young DMD patients with normal EF. LGE may be a useful biomarker for detection of progression of DMD-associated cardiac disease. As such, it may have important implications to clinical care and research.

## Funding

None.

